# Development of Graves orbitopathy after thyroidectomy for thyroid cancer

**DOI:** 10.1210/jcemcr/luag078

**Published:** 2026-04-29

**Authors:** Maria Hormiz, Lochlan Sydenham-Clarke, Noor Lammoza, Jeffrey D Zajac, Mathis Grossmann, Lik Hui Lau

**Affiliations:** Department of Endocrinology, Austin Health, Melbourne, VIC 3084, Australia; Department of Endocrinology, Austin Health, Melbourne, VIC 3084, Australia; Department of General Medicine, Alfred Health, Melbourne, VIC 3004, Australia; Department of Endocrinology, Austin Health, Melbourne, VIC 3084, Australia; Department of Medicine, Melbourne Medical School, University of Melbourne, Melbourne, VIC 3010, Australia; Department of Endocrinology, Austin Health, Melbourne, VIC 3084, Australia; Department of Medicine, Melbourne Medical School, University of Melbourne, Melbourne, VIC 3010, Australia; Department of Endocrinology, Northern Health, Melbourne, VIC 3076, Australia

**Keywords:** Graves disease, Graves orbitopathy, papillary thyroid cancer

## Abstract

We present a case of a 76-year-old female with a history of papillary thyroid cancer treated with a total thyroidectomy, central neck dissection, and radioactive iodine (RAI) in 2006. Sixteen years later, she was found to have lung metastases and underwent a treatment dose of RAI. Given no improvement in her thyroglobulin levels, she underwent a third dose of RAI. One week following this, she presented with new ophthalmalgia, conjunctival injection, and binocular diagonal diplopia. She was diagnosed with a complex ophthalmoplegia, and a magnetic resonance imaging scan of her orbits revealed symmetric bilateral exophthalmos and bilateral enlargement of her extraocular muscles suggesting Graves ophthalmopathy. Her TSH receptor antibody was elevated at 10.1 IU/L (reference range, <1.8 IU/L), consistent with de novo Graves disease. We highlight the possibility that an overflow of TSH-receptor antigens following RAI treatment in patients with metastatic thyroid cancer can lead to the formation of TSH-receptor antibodies, presenting as Graves ophthalmopathy many years following total thyroidectomy.

## Introduction

We present a case of a 76-year-old female who developed Graves orbitopathy in the context of distantly treated papillary thyroid cancer (PTC). Our case adds to the limited literature of Graves disease development after treatment for thyroid cancer.

## Case presentation

A 76-year-old female presented to our hospital in 2022 with acute cholecystitis and was demonstrated to have an incident finding of an 11 × 8 mm lingula lobe nodule on computed tomography (CT). Her medical history was significant for locally invasive PTC diagnosed initially in 2006. She underwent a total thyroidectomy and central neck dissection with 2 positive cervical chain lymph nodes. Histopathology revealed a multifocal PTC with extrathyroidal extension into the sternocleidomastoid. Postoperatively, she underwent 100 millicuries (mCi) of adjuvant radioactive iodine (RAI). The postablation RAI scan revealed a small focus of tracer activity within the thyroglossal tract without evidence of distant metastasis. Other comorbidities included non-insulin–dependent type II diabetes mellitus, obesity, osteoporosis, and a prior 30-pack/year smoking history. She was commenced on 150 mcg of levothyroxine daily.

## Diagnostic assessment

A repeat chest CT 3 months later demonstrated slight growth of the lingula nodule (11 × 10 mm) with new subcentimeter nodules detected throughout both lung fields. A whole body fluorodeoxyglucose (FDG)-positron emission tomography scan demonstrated moderate FDG-avidity within the lingula nodule. A diagnostic thoracoscopic left lower lobe pulmonary wedge resection in March 2022 confirmed metastatic PTC on histopathology.

**Figure 1:**
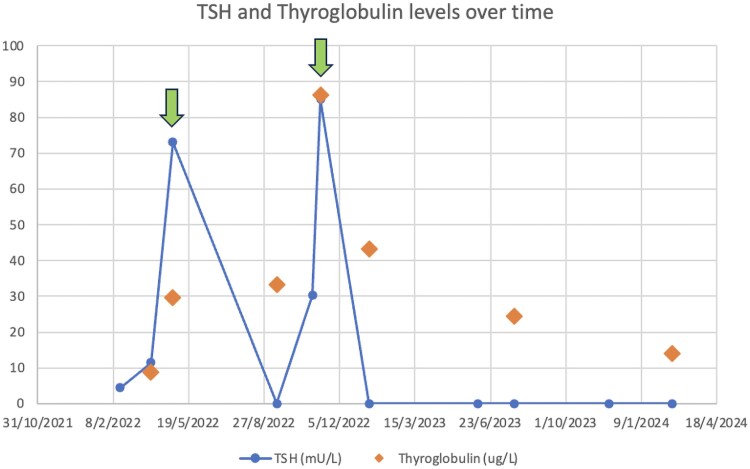
TSH and thyroglobulin levels over time. Arrows show times at which thyroxine hormone withdrawal occurred in preparation for radioactive iodine therapy.

She received 126.2 mCi of adjuvant RAI, with a postablation whole body RAI scan showing no avid recurrent regional nodal or distant metastatic spread; however, persistently elevated thyroglobulin levels 4 months following treatment indicated residual disease (Fig. 1).

Four months following the second dose of RAI, she presented with bilateral periorbital edema. Apart from an orbital CT demonstrating bilateral lacrimal gland prominence, an opthalmological examination was unremarkable, and the patient was discharged for ongoing thyroid cancer surveillance.

Due to a persistently elevated thyroglobulin level of 33.2 μg/L (33.2 ng/mL) (reference range, 1.60-50.0 μg/L; 1.60-50.0 ng/mL) concerning for residual disease, she was treated with a third dose of 148.7 mCi of RAI despite the absence of iodine uptake on a RAI scan. At this time, her TSH was 0.02 mIU/L (reference range, 0.4-4.0 mIU/L). Her postablation scan again revealed noniodine-avid pulmonary nodules. At this time, a repeat FDG-positron emission tomography scan showed a mix of FDG-avid and non-FDG-avid subcentimeter pulmonary nodules, thought to represent undifferentiated/poorly differentiated metastatic PTC. Serial surveillance CT scans at 3, 9, and 18 months following her last RAI dose revealed stable pulmonary nodules with no extrapulmonary disease, and her levothyroxine doses have remained stable at 100 to 150 mcg per day.

**Figure 2:**
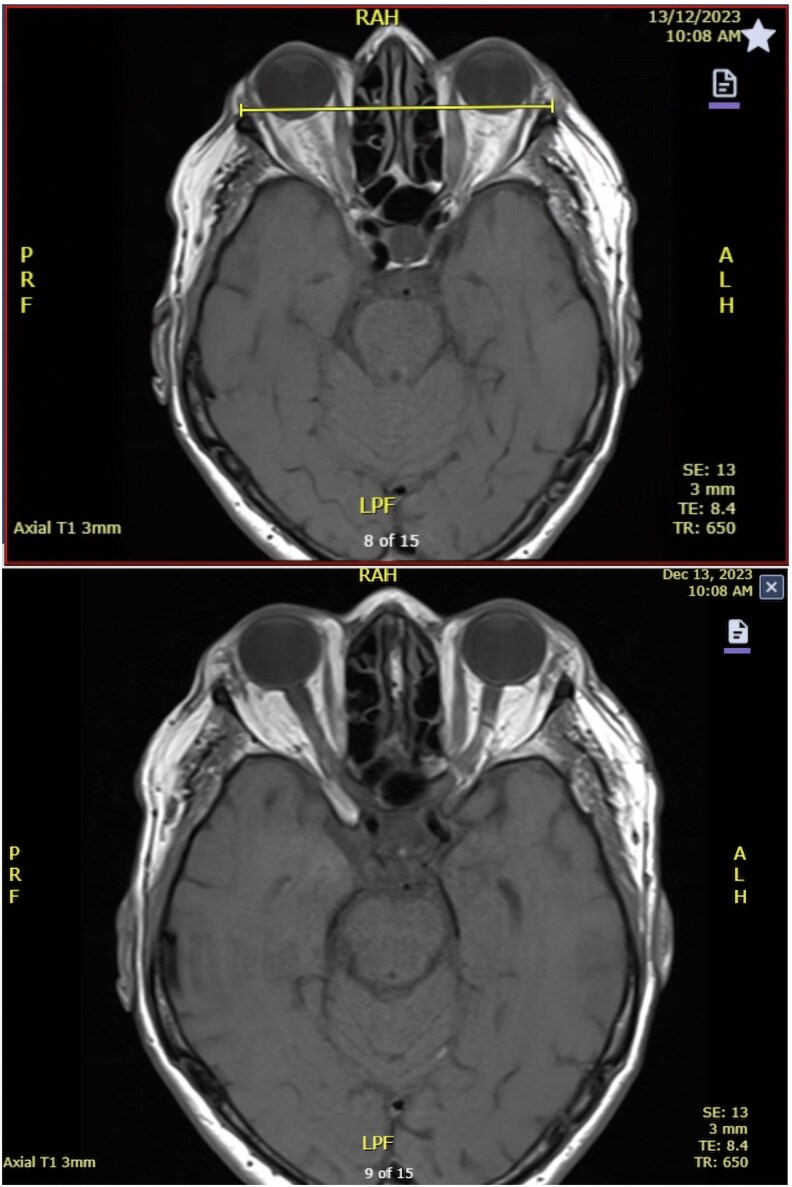
Axial magnetic resonance imaging showing symmetric bilateral exophthalmos and bilateral enlargement of the extraocular muscles, most prominently involving the inferior and medial rectus muscles.

One week after her third dose of RAI, she presented with worsening ocular symptoms, including new ophthalmalgia, conjunctival injection, and binocular diagonal diplopia, and a diagnosis of complex ophthalmoplegia was made. An orbital magnetic resonance imaging showed symmetric bilateral exophthalmos and bilateral enlargement of her extraocular muscles, most prominently involving the inferior and medial rectus muscles, in keeping with Graves ophthalmopathy (Fig. 2). Thyroid-stimulating hormone-receptor antibodies (TRAb) were performed and were significantly elevated (10.1 IU/L) (reference range, <1.8 IU/L). TRAb had not previously been measured in this patient. She was diagnosed with moderate Graves ophthalmopathy with a clinical activity score (CAS) of 3.

## Treatment

Taking into consideration her CAS and underlying comorbidities, the decision was made by the ophthalmology team to avoid upfront systemic glucocorticoids, and treatment with topical lubricating eye drops and oral selenium was initiated.

## Outcome and follow-up

**Figure 3:**
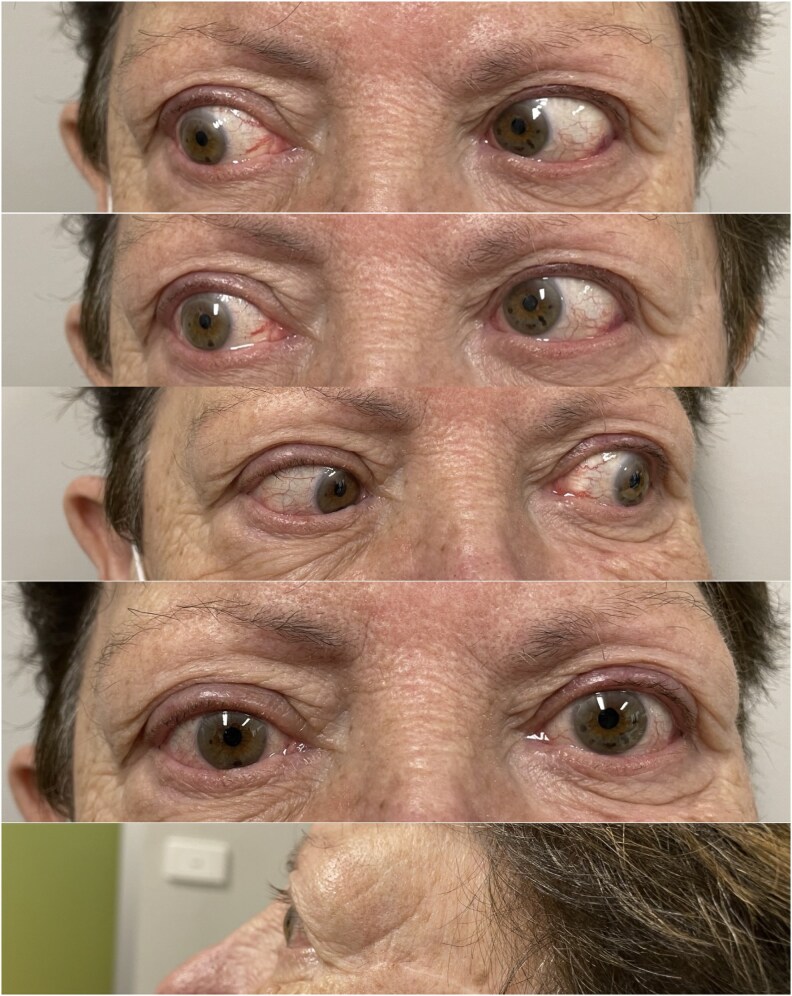
Improvement in exophthalmos but ongoing ophthalmoplegia most prominently on right lateral gaze. Images taken and included with the patient's consent.

On subsequent ophthalmological review, her exophthalmos and conjunctival injection had improved, although her ophthalmoplegia and diplopia persisted (Fig. 3).

## Discussion

Graves disease is the most common cause of hyperthyroidism [1], and approximately 50% of patients develop clinically significant Graves ophthalmopathy. Cigarette smoking is a well-established risk factor for the development of ophthalmopathy, and severe eye disease occurs in 3% to 5% of cases. Risk factors specifically associated with severe ophthalmopathy include male sex and treatment with RAI [2]. The pathophysiology of Graves ophthalmopathy is thought to be driven by the action of IGF-1 receptor antibodies and TRAb on fibroblasts, resulting in immune-mediated inflammation of extraocular muscles, intraorbital fat, and lacrimal glands, which can result in proptosis, diplopia, and rarely optic nerve compression [1].

While RAI is an established therapy for Graves disease and moderate- to high-risk thyroid carcinoma, it has been implicated in the development of de novo Graves ophthalmopathy. The postulated mechanism is the autoimmune production of TRAb against thyroid antigen released following thyroid tissue destruction after RAI exposure [3]. TRAb titers are strongly positively correlated with increased CAS and severity of Graves ophthalmopathy [4]. In our patient, despite the lack of RAI uptake on her postablation whole body scan, we speculate that there was a small volume of overflow of thyroid antigens from her PTC metastases following RAI treatment, leading to the development of TRAb almost 2 decades following total thyroidectomy.

The role of TRAb in stimulating the growth of metastatic thyroid cancer remains uncertain. Current guidelines recommend TSH suppression in patients with high-risk thyroid cancer [5] as TSH causes thyroid hyperplasia via increasing the production of intracellular cAMP [1]. In Graves disease, TRAb causes thyroid hyperplasia via a similar mechanism [1]. In vitro studies of thyroid carcinoma have shown that IgG from the serum of a patient with TRAb increased the concentration of cancer tissue cAMP [6], suggesting TRAb may also be implicated in the growth of thyroid carcinoma. Toda et al [7] have suggested testing for TRAb in patients with progressive metastatic thyroid carcinoma despite suppressive TSH therapy [7]. However, there are currently no targeted TRAb-lowering therapies available [8].

To identify further comparable cases, a literature review was conducted using OVID-Medline from the date of inception until August 2024, using the search terms “thyroid carcinoma,” “Graves disease,” and “thyroidectomy.” An updated bridging review was performed in March 2025. This yielded 16 case reports of patients who developed TRAb positivity following total thyroidectomy for thyroid cancer [6-22]. Of these, 8 cases were PTC and 8 follicular. The median age at thyroid cancer diagnosis was 54.5 years (interquartile range 49-57.5). TRAb positivity emerged a median of 6 years after thyroidectomy (range: 3 months to 10 years) and a median of 4 years after the last RAI treatment (range: 2 weeks to 10 years). Metastatic lesions varied with regard to iodine avidity, with thyroid eye disease also variably present across cases [9-16, 18, 19, 22]. Importantly, clinical thyrotoxicosis was observed exclusively in patients with iodine-avid metastatic disease [6, 7, 11, 17, 20-22], likely due to TRAb interacting with the functioning TSH receptor on metastatic thyroid tissue. Management of thyrotoxicosis typically involved further RAI treatment, supplemented by antithyroid medications in some cases. In contrast, our patient had no iodine-avid metastases and did not exhibit clinical features of thyrotoxicosis. We hypothesize this to be due to a defect in the activation of the TSH receptor no longer engaging the sodium-iodine symporter [23]. Table 1 provides a comprehensive summary of the individual cases.

**Table 1:** 16 cases reported in the literature of TRAb positivity and manifestations of Graves disease after total thyroidectomy and RAI treatment

Author, year of publication	Age at thyroid cancer diagnosis, sex	Histopathology	Time to TRAb positivity after thyroidectomy	TRAb/TSI titre	Number of RAI treatments and cumulative dose	Time to positive TRAb after most recent RAI	Iodine-avid metastases	Thyrotoxicosis and treatment	Presence of eye disease
Deguchi-Horiuchi et al, 2024 [9]	56, female	Papillary	9 years	TRAb 8.38 IU/L (ref. 0-2 IU/L)	2, 150 mCi	4 years	Initially yes, then no	No	Yes, triamcinolone injection
Toda et al, 2022 [7]	51, male	Follicular	7.5 years	TRAb 28.3 IU/L (ref. 0-1.9 IU/L)	1, 100 mCi	7.5 years	Initially no, then yes	Yes, methimazole	NA
Zgubienski et al, 2020 [10]	49, female	Papillary	3 months	TRAb 40 IU/L (ref. 0-1 IU/L)	1, 5 mCi	3 months	No	No	Orbitopathy, classic MRI features. Treated with methylprednisolone
Krishnaja et al, 2019 [11]	63, female	Follicular	2 years	NA	1, 200 mCi	TRAb preceded RAI	Yes	Yes, RAI	Prominent eyes, staring
Aoyama et al, 2017 [6]	57, female	Follicular	4 years	TRAb 20 IU/L (ref. 0-1.5 IU/L)	5, 500 mCi	“soon after”	Yes, remained iodine-avid	Yes, methimazole + 100 mCi RAI	NA
Jang et al, 2015 [12]	49, female	Papillary	27 months	TSI 441.4 SRR% (ref. 0-140 SRR%)	1, 30 mCi	25 months	No mets	No	Yes, triamcinolone injection
Giovansili et al, 2011 [13]	53, female	Papillary	5 years	TRAb 8 IU/L (ref .< 1 IU/L)	1, 100 mCi	4 years	No mets	No	Yes
Woeber et al, 2008 [14]	44, female	Papillary	7 years	TSI 290% (ref. < 130%)	3, 350 mCi	1 month	No	No	Yes
Antonelli et al, 2008 [15]	58, female	Papillary	1 year	TRAb 6 IU/L	1, 100 mCi	1 year	No mets	No	Yes
Berg et al, 2005 [16]	57, female	Papillary	4 years	TRAb 412 U/L (ref. 0-9 U/L)	3, not specified	2 weeks	No	No	Yes, prednisolone then methimazole
Basaria and Salvatori, 2002 [17]	64, male	Papillary	10 years	TSI 143%	3, 523 mCi	TRAb positivity preceded RAI	Yes, remained iodine-avid	Yes, propylthiouracil and RAI	NA
Cobin, 2000 [18]	62, female	Follicular	4 years	TSI 99% (ref. < 15%)	1, 30 mCi	4 years	No	No	Yes, orbital decompression
Katz et al, 1997 [19]	49, female	Follicular	9 years	TSI 823% (ref. < 130%)	2, 250 mCi	9 years	No	No	Yes, methimazole, RAI, corticosteroids, and orbital radiotherapy
Yoshimura et al, 1997 [20]	53, male	Follicular	8 years	TSAb 176% (ref .< 145%)	2, 200 mCi	6 years	Yes, remained iodine-avid	Yes, 100 mCi RAI + methimazole + inorganic iodine + dexamethasone	No
Kasagi et al, 1994 [21]	48, female	Follicular	9 months	TSAb 150% (ref. 55-145%)	7, 714.8 mCi	7 months	Yes	Yes, RAI	No
Snow et al, 1979 [22]	57, male	Follicular	10 years	TSAb 14% (ref. > 72%)	4, 425 mCi	10 years	Yes	Yes, RAI	Yes, RAI

Abbreviations: mets, metastatic disease; MRI, magnetic resonance imaging; NA, not available; RAI, radioactive iodine; ref., reference range; SRR, specimen-to-reference ratio; TSAb, thyroid-stimulating antibody; TSI, thyroid-stimulating immunoglobulin.

Our case, alongside existing literature, demonstrates that Graves disease can develop in patients receiving RAI for thyroid carcinoma after thyroidectomy, albeit rarely. The heterogeneity in clinical presentation makes it challenging to identify which patients may be at risk and may benefit from screening. While the current American Thyroid Association guidelines do not suggest screening for concurrent undiagnosed Graves disease in patients with thyroid cancer [5], any ocular symptoms in patients post-RAI therapy should prompt clinicians to consider concurrent Graves disease, especially in patients with other risk factors. Clinicians should also be aware that TRAb positivity can manifest years after thyroidectomy [20, 21].

## Learning points

- Overflow of TSH-receptor antigens following RAI administration can lead to an increase in TRAb titers, a known risk factor for thyroid eye disease.
- Graves eye disease can present many years following thyroidectomy and should be considered a differential diagnosis for patients presenting with ophthalmopathy post-RAI administration.
- TRAb may be implicated in the growth of thyroid metastases; however, the lowering of antibody titers is not part of the standard of care. This represents an area of uncertainty within current clinical practice.
- Metastatic thyroid carcinoma with positive TRAbs in patients presenting with postthyroidectomy thyrotoxicosis should be considered.

## Contributors

All authors made individual contributions to authorship. M.H., N.L., and L.S.C. were involved in the writing of the manuscript, literature review, and figure and table generation. J.D.Z., M.G., and L.H.L. were involved in the diagnosis and management of this patient and writing and providing expertise and guidance to the manuscript.

## Data Availability

Some or all datasets generated during and/or analyzed during the current study are not publicly available but are available from the corresponding author on reasonable request.
